# In-Situ SERS Detection of Hg^2+^/Cd^2+^ and Congo Red Adsorption Using Spiral CNTs/Brass Nails

**DOI:** 10.3390/nano12213778

**Published:** 2022-10-26

**Authors:** Mohamed Shaban

**Affiliations:** Department of Physics, Faculty of Science, Islamic University of Madinah, Madinah 42351, Saudi Arabia; mssfadel@aucegypt.edu

**Keywords:** spiral CNTs, heavy metals, sensors, nanoadsorbents, congo red dye adsorption

## Abstract

Brass spiral nails were functionalized with CoFe_2_O_4_ nanoparticles and utilized as a substrate for the growth of extremely long CNTs with helical structures and diameters smaller than 20 nm. Different methods were used to characterize the grown CNTs’ structures and morphologies. The characteristic Raman peaks of CNTs were amplified four times after being uploaded on the spiral nail, making the substrates for surface-enhanced Raman spectroscopy (SERS) more sensitive. To detect Hg^2+^ and Cd^2+^ at concentrations ranging from 1 to 1000 ppb, a CNT/spiral brass nail was used as a SERS substrate. The proposed sensor demonstrated high sensitivity and selectivity between these heavy metal ions. As a result, the proposed CNTs/spiral brass sensor can be an effective tool for identifying heavy metal ions in aqueous solutions. In addition, Congo red (CR) adsorption as a function of initial dye concentration and contact time was investigated. For CR dye solutions with concentrations of 5, 10, and 20 mg/L, respectively, the highest removal percentage was determined to be ~99.9%, 85%, and 77%. According to the kinetics investigation, the pseudo-first-order and pseudo-second-order models effectively handle CR adsorption onto CNTs/spiral nails. The increase in the dye concentration from 5 ppm to 20 ppm causes the rate constant to drop from 0.053 to 0.040 min^−1^. Therefore, our sample can be employed for both the effective degradation of CR dye from wastewater and the detection of heavy metals.

## 1. Introduction

A significant global challenge is the removal of heavy metals and dyes from water sources. To remove dyes and heavy metals, many technologies have been developed, including the low-cost adsorption technique using different adsorbents such as layered double hydroxides, clays, zeolites, and carbon-based nanoadsorbents [1,2,3,4,5]. Carbon nanotubes (CNTs) are attracting a lot of attention because of their distinctive optoelectronic applications in flat panel displays, very sensitive electromechanical and chemical sensors, and hydrogen storage devices [6,7,8,9]. CNTs have also been used to remove harmful pollutants from gas streams and aqueous solutions because of their sizable specific surface area, highly hollow and porous structure, low mass density, and effective interactions with pollutant molecules [10,11]. Several experimental studies on the adsorption of heavy metal ions on single- and multi-wall CNTs have already been conducted [10,12,13]. Carbon nanotubes (CNTs) are a typical adsorbent used to extract organic and other inorganic pollutants from wastewater [14,15,16]. CNTs are used as adsorbents more frequently than conventional adsorbents due to their superior volume-area ratios, functional surfaces, strong electrostatic interactions, cylindrical hollow structures, high mechanical strengths, and quicker equilibrium durations [17,18,19]. Single-walled carbon nanotubes (SWCNTs) and multi-walled carbon nanotubes are the two varieties of CNTs (MWCNTs). By enhancing interactions with organic dye molecules and heavy metals through hydrophobic surfaces, electrostatic forces, van der Waals forces, and hydrogen bonding, both of their structural configurations are more effective [20]. MWCNTs are also superior to SWCNTs at removing cationic dyes because of interactions between charged dye molecules and their surface [15]. So far, MWCNTs have only been used for CR removal in a small number of experiments that showed strong adsorption performance [21,22,23,24].

Additionally, by guiding, amplifying, emitting, and altering optical fields, CNTs placed on substrates can be used for novel and efficient applications, such as sensors based on SERS [25,26,27,28]. SERS is a technique that enhances Raman scattering by molecules adsorbed onto particular, meticulously created rough metal surfaces. The SERS-based sensor is one of the most important nanooptical-based detection methods for the chemical speciation of hazardous heavy metal ions in water at trace levels. Li et al. [29] evaluated and offered a summary of the successes and challenges in the determination of heavy metals, organic pollutants, ions, and pathogens based on SERS. A porous anodic alumina membrane functionalized with hexagonal arrays of Au nanoparticles was developed by Shaban et al. [30,31,32] as a SERS substrate for the detection of several heavy metals, including Hg^2+^, Cd^2+^, and Pb^2+^ at nM concentrations. To create highly sensitive SERS biosensors for the detection of organic or inorganic contaminants and diseases, Wei et al. [33] compiled the uses of plasmonic nanoparticles. Chemical and electromagnetic enhancements are the two processes for SERS enhancement that are discussed in the literature. The chemical effect is improving by two orders of magnitude, while the electromagnetic (EM) effect dominates. The high costs and reproducibility of sensor fabrication continue to hinder the practical environmental applications of SERS-based sensors despite their rapid growth. There is still a high demand for the creation of quick, cheap, repeatable, and scalable detection platforms.

Numerous methods, including arc discharge, laser ablation, plasma enhancement, and chemical vapour deposition (CVD) of hydrocarbon gasses (methane, ethane, and acetylene) at relatively high temperatures over a catalytic material, have been used to create CNTs [34,35]. The band gap of semiconducting nanotubes can be adjusted by varying the tube diameter, and the deposited CNTs may be made of metals, semiconductors, or dielectrics [36]. Along with MWCNTs, amorphous carbon is also present during the creation of MWCNTs. These amorphous carbons are unwanted in many applications; thus, they are etched out of the structure. Another problem is the need for unique methods to remove the nanoadsorbent from the aqueous solution after usage. Additionally, finding novel methods to produce MWCNTs at high yields is crucial. In order to address these problems, it is suggested in this work that a coating of CNTs with a diameter no greater than 20 nm be applied to the spiral nail’s upper surface. As synthetic materials for the SERS-based detection of heavy metals and the adsorption of the CR dye from aqueous solutions, the developed MWCNTs/spiral brass nails are used. Hg^2+^ and Cd^2+^ were chosen as the test objects due to their numerous detrimental effects on human health as well as the economy. Therefore, the continuous detection and monitoring of these harmful heavy metal levels in soil, pharmaceuticals, and water resources require the development of rapid, trustworthy, and sensitive sensor networks [37,38,39,40]. Processing speed, operation costs, product yield, reusability, and sustainability all benefit from this. To detect traces of Hg^2+^ and Cd^2+^ ions in water, CNTs and spiral brass nails are made. The in-situ user’s spectroscopy serves as the cornerstone of the sensing principle. Research is being conducted on sensitivity and selectivity. The use of the samples for the adsorption of CR is also being explored, as=is the impact of the initial dye concentration and adsorption period. The mechanics of the reaction and its tenfold repeatability are also highlighted.

## 2. Methods

### 2.1. Functionalization of Spiral Nail with CoFe_2_O_4_ Nanoparticles

Brass spiral nails were cleaned, made functional, and utilized to generate CNTs in high yield. The spiral nail was cleaned and functionalized using CoFe_2_O_4_ nanoparticles. A 1:1 mixture of 0.5 M Fe(NO_3_) and 0.5 M Co(NO_3_)_2_·6H_2_O was created. The cleaned nail was then submerged in the solution and placed in the ultrasonic for one hour. The produced nail was dried for one hour in an oven at 500 °C.

### 2.2. Growth of CNTs on Functionalized Nail

In a ceramic tube with a temperature and gas flow controller, C_2_H_2_ chemical vapour deposition was performed (CVD). Spiral brass nails functionalized with CoFe_2_O_4_ were heated to 600 °C for 10 min while being exposed to nitrogen (N_2_) gas. The system was then subjected to a mixture of N_2_/C_2_H_2_ (5:1 *v*/*v*) for 50 min. The tube furnace’s temperature decreased as the N_2_ gas passed through the reaction chamber. In a 120 °C oven, the prepared CNTs/spiral brass nails were cured.

### 2.3. Characterization Techniques

The produced nanostructured films underwent morphological studies using field emission-scanning electron microscopy, or FE-SEM (model: ZEISS SUPRA 55 VP and ZEISS LEO, Gemini Column, Jena, Germany). Transmission electron microscopes (TEM, JEOL-2010F, Tokyo, Japan) were also used to analyze the interior morphology. Research of the chemical composition was performed using energy-dispersive X-ray analysis (EDX; Oxford Link ISIS 300 EDX, Oxford, UK). To produce an accurate quantitative analysis, the reaction was calibrated using standard additions.

### 2.4. SERS Measurements

The sensing approach is based on heavy metal detection utilizing surface-enhanced Raman scattering (SERS) spectroscopy after injecting very small amounts (0.1 l) of water polluted with heavy metals on the surface. Using an Enwave Optronics Raman microscope with a 514 nm excitation wavelength and a 1 μm spot size, SERS measurements were made on CNT and nail samples. P = 50 mW and t = 20 s were the excitation laser power and exposure times, respectively.

### 2.5. Adsorption Measurements

Adsorption studies were performed on CNTs/spiral brass nail adsorbents, utilizing a variety of starting dye concentrations and adsorption periods. With a variety of experimental conditions, including starting dye concentrations (5–20 mg/L), adsorption periods (0–90 min), adsorbent dose (0.05 g per 50 mL of CR solution), pH (7.0), and room temperature, all CR adsorption tests were conducted on a batch mode scale. The UV/Vis spectrophotometer (PerkinElmer, Lambda 950, Boston, MA, USA) was used to determine the variance in CR concentration by tracing the absorption peak. With 0.05 g in 50 mL of initial CR concentration of 5 mg/L for 90 min of contact time at room temperature and pH 7, the adsorbent’s reusability was examined ten times. After each run, the adsorbent was washed with DI water and set for the next run.

The CR dye elimination percentage was calculated using Equation (1) [41,42]:

(1) $$\;\mathrm{CR}\;\mathrm{dye}\;\mathrm{removal}\;\%=\frac{{\left(C_{o}-C_{t}\right)}_{}}{C_{o}}\times 100\;\;$$

where *C_o_* and *C_t_* are, respectively, the starting and final concentrations of CR in mg/L. The data that were displayed were the averages from three separate studies.

## 3. Results and Discussions

### 3.1. Morphological Study

**Figure 1:**
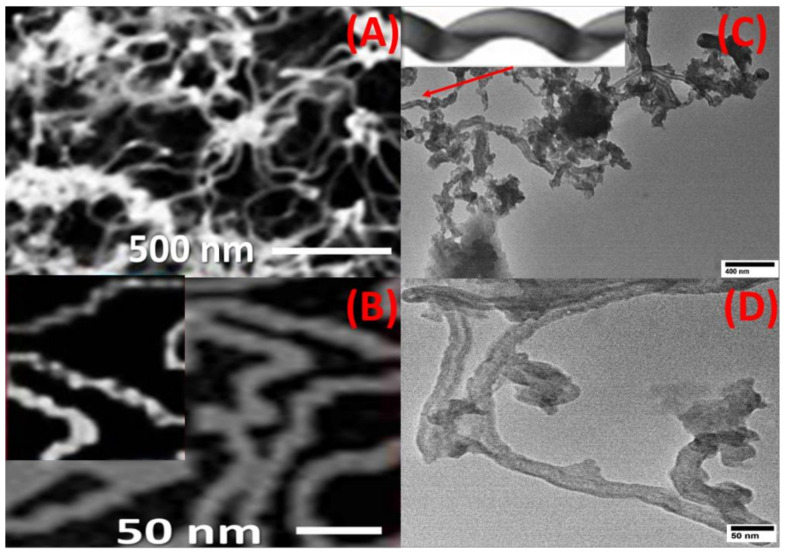
Top view (**A**,**B**) FE-SEM and (**C**,**D**) TEM image of CNTs/spiral nail. The inset of (**C**) shows the helical shape of one CNT.

CNTs were grown on the top surface of the spiral nail using CVD to enhance its sensing and adsorbent capabilities. By using FE-SEM and HR-TEM images, the surface morphology of the CNT-coated spiral nail was identified. Top-view HRTEM and FE-SEM images of CNTs at various magnifications are shown in Figure 1A–D. The CoFe_2_O_4_ nanoparticles on the top surface of the spiral have served as seeds for growing extremely long CNTs with diameters of 17 ± 3 nm, as seen in SEM and TEM pictures, Figure 1A,C. Helix-shaped carbon nanotubes with a sizable specific surface area were displayed in Figure 1B,C. One CNT’s helical shape is depicted in the inset of Figure 1C. The inner diameter was 8 ± 2 nm and the CNT wall width was 8 ± 1 nm, according to Figure 1D.

**Figure 2:**
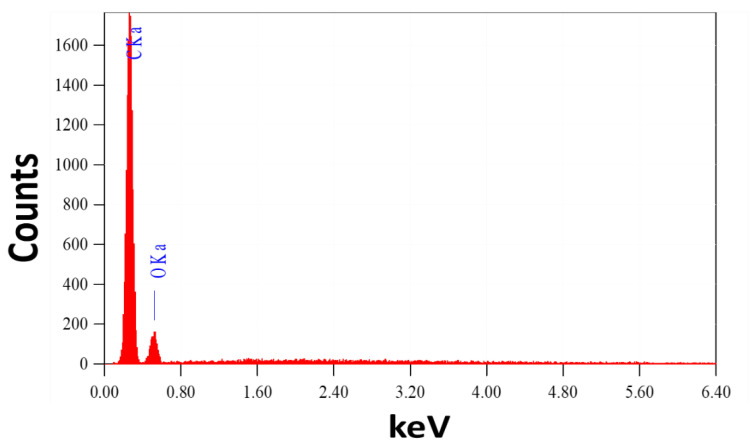
EDX spectrum of CNTs powder etched from the surface of the brass nail.

It is vital to note that this process yields about 17 g of CNTs. The great stability of the manufactured helical CNTs on the spiral brass nail, in addition to their low cost and high yield, makes these innovative nanostructures promising candidates for SERS substrates and may find a wide range of new applications, such as in sensors and environmental monitoring. It can also be utilized as a potential nano-adsorbent nail for the purification of water. Energy-dispersive X-ray (EDX) analysis of the chemical makeup of the mechanically etched CNTs is used to confirm the purity of the prepared CNTs, as shown in Figure 2. The signals of the elements C and O are seen in the CNT powder’s EDX spectrum. From this EDX chart, no impurity signs have been identified.

### 3.2. Raman Spectra

**Figure 3:**
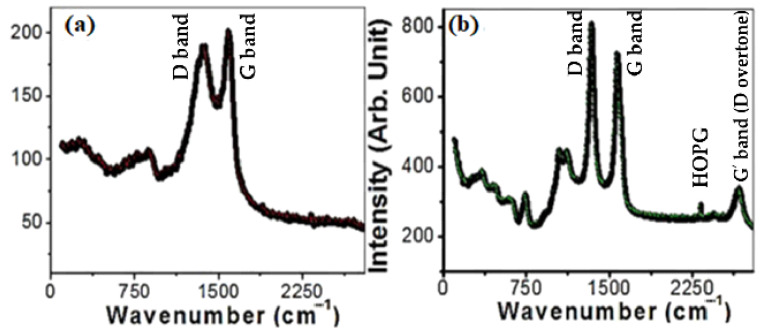
Raman spectra of (**a**) CNTs powder and (**b**) CNTs/ spiral nail.

The Raman spectra of MWCNTs and MWCNTs loaded on spiral nails are displayed in Figure 3. Three distinctive peaks are depicted at 870, 1370, and 1590 cm^−1^, as seen in Figure 3a. As illustrated in Figure 3b, two prominent first-order characteristic peaks for MWCNTs were found at 1573 cm^−1^ (G band) and 1341 cm^−1^ (D band). The G band represents the E_2g_ phonons, whereas the D band is the A_1g_ symmetry breathing modes of rings or K-point phonons [43]. Because the nanotubes are graphitic, the G band is connected to the in-plane stretching vibrations of sp2-bonded carbon atoms [43]. Defects and disorders in the hexagonal lattice, such as carbonaceous impurities and broken sp2 bonds in the sidewalls, are associated with the D band [44]. The graphitic lattice of the system is measured by the D/G intensity ratio (ID/IG), which also reflects how faulty it is. The calculated I_D_/I_G_ ratio is 0.955. In addition, the G’ band (D overtone or 2D-band) was observed at ~2670 cm^−1^ in Figure 3b. The weak band at 2317 cm^−1^ is ascribed to highly oriented pyrolytic graphite (HOPG). The weak bands observed before 1120 cm^−1^ are related to the vibrational modes of the used brass nail and used catalyst. The peaks at ~1120 and 1041 cm^−1^ are attributed to multi-phonon transitions of brass [45]. Additionally, four peaks at 740 (A_1g_(1)), 615(A_1g_(2)), 467 T_2g_(2), and 340 (E_g_) cm^−1^ are related to the Raman active modes of CoFe_2_O_4_ [46,47].

All the peaks are moved to lower wavenumbers by uploading the CNTs on the spiral nail (Figure 3b). This change suggests some decreases at the CNT/spiral nail interfaces during CNT growth in a high-temperature N_2_ environment [48]. Additionally, the ID/IG value rising to 1.12 suggests that the disorders and flaws of CNTs on spiral nail surfaces are more severe than those of free-standing CNTs. The low crystallinity of the CNTs is shown by the high intensity of the D band. Three factors make the deposition of very long helical CNTs on the spiral nail’s outer surface crucial for sensing applications. The CNTs on the spiral’s outer surface have a greater chance of coming into contact with the reactant, to start. Second, the increased surface area of CNTs and the spiral nail membrane, along with the improved Raman characteristics, might improve the sensing properties. Last but not least, the presence of abnormalities and illnesses may function as additional hot spots. For these reasons, this CNT/spiral nail array SERS substrate is intriguing.

### 3.3. Sensing Properties and SERS Measurements

**Figure 4:**
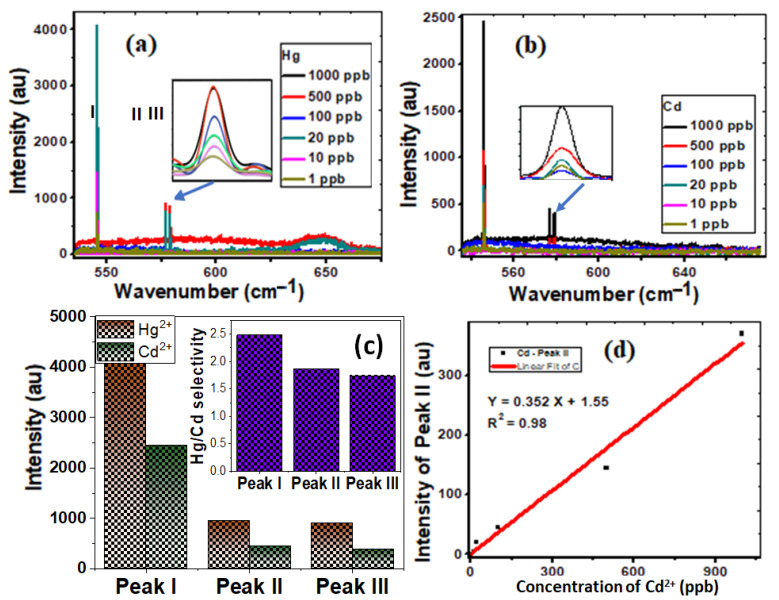
Demonstrating the sensitivity and selectivity of spiral CNT/brass nails uploaded with (**a**) Hg^2+^ and (**b**) Cd^2+^ of concentration 1–1000 ppb; (**c**) the variation of intensity of the characteristic peaks, I, II, and III, for Hg^2+^ and Cd^2+^ ions; (**d**) the linear variation of intensity of the characteristic peak II for Cd^2+^. The inset of (**c**) shows the selectivity of Hg^2+^ ions over Cd^2+^ ions.

Surface-sensitive Raman scattering (SERS) is a technique that makes molecules adsorbed on rough metal surfaces boost Raman scattering. SERS measurements are used to investigate the vibrational properties of the adsorbed molecules, revealing structural information about the molecule and its local relationships. SERS measurements, therefore, allow for and specifically identify individual detection [49,50]. The unique properties of CNTs/spiral nail nanostructures enable high sensitivity and selectivity in a novel manner. Figure 4a,b display the SERS spectra of CNT/spiral nails contaminated with 1–1000 ppb of Hg^2+^ and Cd^2+^. The Raman Hg peaks are more intense than their Cd counterparts. Additionally, as shown in the inset of Figure 4b, increasing the detected ions’ concentration enhances the contrast and intensities of the SERS peaks. Figure 4c shows the fluctuation of the peak I, II, and peak III intensities at 1000 ppb of Hg^2+^ and Cd^2+^. Peak I exhibits the biggest change in intensity. The inset of Figure 4c displays the selectivity of Hg^2+^ over Cd^2+^ ions using I_Hg_/(I_Hg_ − I_Cd_). The corresponding values for the three peaks were 2.5, 1.9, and 1.7. In addition, the presence of Cd^2+^ ions boosts the strength of peak II (Figure 4d).

As the concentration rises from 1 to 1000 ppb, the intensity of peak II for Cd increases from 1 to 370. According to Figure 4d, there was a linear relationship between the peak II values’ intensity and the concentrations of Cd^2+^ ions. Since the curve is linear, Equation (2) provides a good match:

Y = 0.352 X + 1.55,      (R^2^ = 0.98) (2)

This may be related to the chemical modification of the spiral nail’s surface and more potent hotspots, which are active nanodots with diameters of less than 20 nm [30,51]. As a result, compared to Cd^2+^ ions, Hg^2+^ ions were more sensitive and selective. The interference of the reflected rays from the air/metal contacts may also lead to electromagnetic enhancement and significant signal amplification [51]. The trapping of CNT with adsorbed heavy metals then enhances the sensing performance because of the increase in the number of analyte molecules in SERS-active hot spots and the electromagnetic amplification caused by the interference of optical signals inside the interaction volume. Raw CNTs are infrequently employed for the selective sorption of ion metals because of the van der Waals interactions between the carbon atoms in graphene sheets. The functionalization of CNTs improves medium dispersion while also improving metal ion sorption through chemical bonding, making modified CNTs better sorbents and more selective for metal ions than raw CNTs [52]. Helical carbon nanotubes with a large specific surface area have been used to successfully detect heavy metals on the spiral nail’s upper surface. The developed spiral CNT/brass nail is tested for ten cycles of Hg^2+^ ion detection, and the results are shown in Figure S1 (Supplementary Materials), along with the descriptive statistics results in the inset table. The results confirm the high reusability of the designed SERS sensor.

### 3.4. Congo Red Adsorption and Kinetic Models

**Figure 5:**
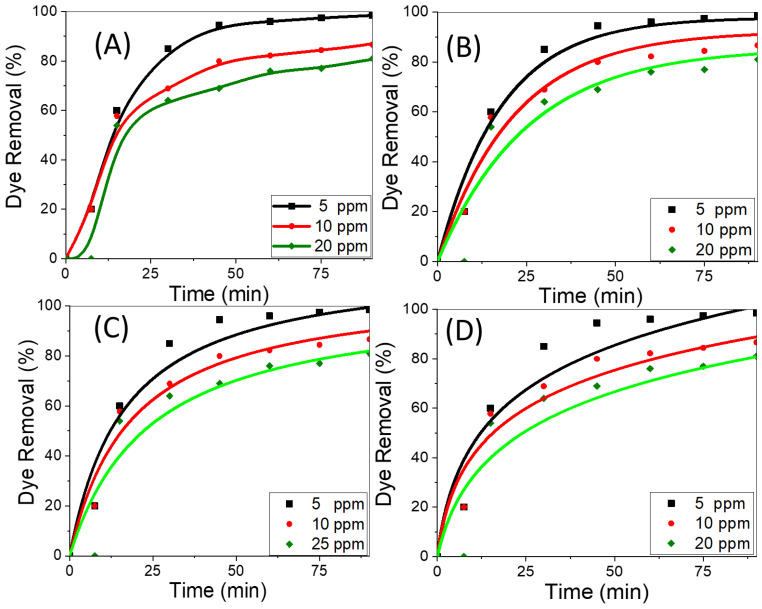
(**A**) Removal percentage versus time at different dye concentrations, (**B**) nonlinear pseudo-first-order, (**C**) nonlinear pseudo-second-order, and (**D**) nonlinear Elovich sorption kinetics of CR dye at 25 °C and pH 7.

The amount of dye removed by adsorption is significantly influenced by the starting concentration of the adsorbate. Using CNT/spiral nail nanoadsorbents at different initial CR concentrations, Figure 5A shows the variations in the removal percentage of CR adsorbed over time. The dye removal percentage normally begins the adsorption process at a relatively high level and then gradually decreases until it achieves equilibrium. Once equilibrium is attained while using fresh sorbents, contact time has no discernible effect on the adsorption process. The reason for the rapid removal rate during the early stages of adsorption progression is the presence of numerous exposed active adsorption patches on the surfaces of the adsorbent. By lengthening the contact time between the adsorbent and the adsorbate, the hot spots were transformed into fully occupied CR sites. As a result, there is an increase in the repulsive forces between CR molecules in the bulk liquid phase and CR molecules adsorbed on adsorbent surfaces [41]. The dye removal percentage falls as the initial CR dye concentration is raised. In contrast, the key factor driving the increase in the amount of adsorbed CR by the adsorbent is the high driving force for mass transfer at a high starting CR concentration. This may be attributed to the gradient’s increase with increasing beginning CR concentration. In order to overcome the mass transfer resistance between the CR adsorbate and the nanoadsorbent active sites, an appropriate rise in the draft forces, therefore, takes place [53,54]. At pH 7 and 25 °C, the highest removal rates for CR dye solutions with concentrations of 5, 10, and 20 mg/L, respectively, were determined to be 99.9%, 85%, and 77% at 90 min. The findings indicated that CNT growth on the surface of spiral nails produced higher values than had previously been observed for CNT-based adsorbents [55,56,57], indicating the viability of the employed strategy to improve CR removal performance, particularly at lower concentrations.

To select the best adsorption kinetics model, the adsorption performance of CNTs/spiral nails at various initial CR concentrations was examined. Previous reports stated that the non-linear forms of kinetic models are more suitable than the linear forms for fitting the experimental data and that nonlinear fitting is more accurate than linear fitting to obtain the values of the constants for each model [58,59,60]. By plotting the proportion of dye removal percentage vs. time, Figure 5B–D depict the first-order, second-order, and Elovich kinetics nonlinear graphs [61,62]. The adsorption kinetics parameters for the assessment model; k_1_, k_2_, qe, β, and α; as well as R^2^, were derived using nonlinear fitting and are displayed in Table 1. The pseudo-first-order and second-order models are effective at handling CR adsorption onto the proposed nanoadsorbent at low and high CR concentrations, respectively, according to the nonlinear fit and regression coefficient values in Table 1 for all the studied kinetic models. This was further supported by the accurate approximation between the calculated dye removal percentage and the experimental removal percentage. According to the first-order model, the rate constant is lowered from 0.053 to 0.040 min^−1^ by raising the dye concentration from 5 ppm to 20 ppm. With the pseudo-first-order model at low concentrations and the pseudo-first-order model at high concentrations, the CR adsorption on CNTs/spiral nails is effectively addressed.

### 3.5. Reusability Test

**Figure 6:**
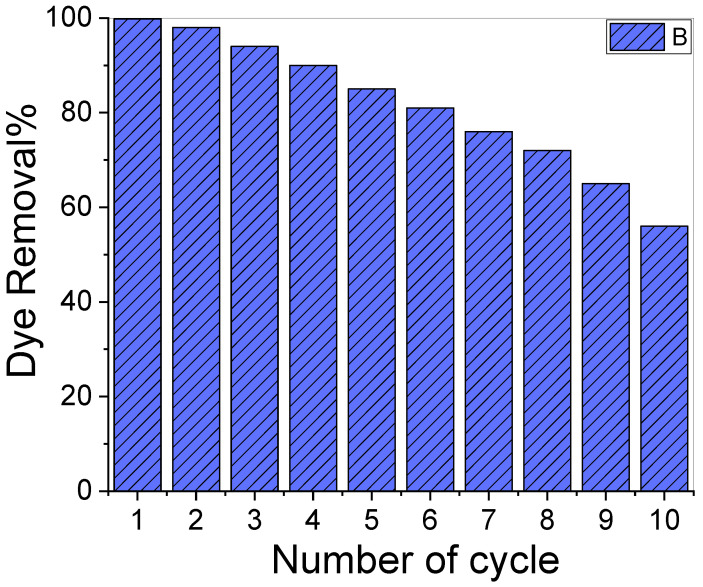
Reusability test of the sample for ten cycles.

Figure 6 illustrates a reusability test for CR elimination using the same adsorbent and dose ten times. The results showed that over the ten adsorption cycles, the removal percentage of the used adsorbent varied significantly. The dye removal percentage for our adsorbent was 99.9% at the beginning of the experiment and decreased to ~56.0% towards the end. The decrease in the CR removal percentage might be attributed to the CR molecules adhering to the adsorbent’s surface, which then blocked the surface from the dissolved CR molecules and resulted in a drop in adsorption capacity [63].

As a result, a new approach was used to produce high-yield CNTs and to produce spiral CNTs/brass nails. The samples that are generated can be used to continually detect and track the levels of harmful heavy metals (Hg^2+^ and Cd^2+^) in pharmaceuticals, food processing, and soil. Additionally, recycling industrial wastewater from the paper, textile, and plastics industries is another application for the developed spiral CNTs/brass nanoadsorbent.

## 4. Conclusions

Here, we introduced a brand-new type of SERS sensor for the detection of heavy metals (Hg^2+^ and Cd^2+^) at ppb concentrations (1–1000) with good sensitivity and selectivity. On the surface of spiral brass nails functionalized with CoFe_2_O_4_ nanoparticles, very long helical CNTs with diameters < 20 nm were generated by CVD. As a more dependable and sensitive heavy metal sensor, CNTs/nail has been proven. The lowest measurable concentration at room temperature in this investigation was 1 ppb, and the CNTs/brass nail sensor demonstrated remarkable sensitivity and selectivity for Hg^2+^ over Cd^2+^. The direct and high sensitivity, selectivity, low cost of production, low cost of monitoring, and simplicity of the suggested CNTs/spiral brass nail sensor are all present. Therefore, a portable Raman spectrometer can use the current sensor as a potent tool to determine the presence of heavy metal ions on-site. The adsorption activity of helical CNTs/spiral brass nails is demonstrated as a function of the starting dye concentration and contact time. For CR dye solutions with concentrations of 5, 10, and 20 mg/L, the highest removal percentage was determined to be 99.9%, 85%, and 77%, respectively. The kinetics analysis reveals that at low and high concentrations, respectively, CR adsorption onto CNTs/spiral nails is successfully explained by the pseudo-first-order and second-order models. The rate constant decreases from 0.053 to 0.040 min^−1^ with an increase in dye concentration from 5 ppm to 20 ppm. Because of this, our sample may be used to effectively degrade CR dye in wastewater and identify heavy metals. Consequently, a brand-new, reasonably priced green nanoadsorbent that would aid in the best industrial wastewater recycling might be produced. The suggested SERS sensor can also be quickly integrated into an automated signal detection platform on a fabrication-friendly sensing platform for further functionality.

## Figures and Tables

**Table 1:** Parameters of the nonlinear kinetic models for CR dye adsorption on the surface of the sample.

**Pseudo-first order: ** Y = q_e_ [1 − exp(−k_1_X)]		
5 ppm	$\mathrm{Rate}\;\mathrm{constant}=k_{1}$ (min^−1^)	0.05335 ± 0.00598
	maximum amount of CR uptake = q_e_ (mg/g)	100.66688 ± 2.67081
	R^2^	0.98094
10 ppm	$k_{1}$	0.04689 ± 0.00559
	q_e_	92.32928 ± 2.43059
	R^2^	0.97063
20 ppm	$k_{1}$	0.03962 ± 0.00817
	q_e_	85.63994 ± 4.24925
	R^2^	0.92628
**Pseudo-second order: ** $Y\;=\frac{k_{2}q_{e}^{2}X}{1+{\;k}_{2}q_{e}\;X}$		
5 ppm	$\mathrm{Rate}\;\mathrm{constant}=k_{2}$(min^−1^)	4.91311 × 10^−4^ ± 1.49132 × 10^−4^
	q_e_ (mg/g)	119.81004 ± 7.39609
	R^2^	0.96082
10 ppm	$k_{2}$	5.02665 × 10^−4^ ± 9.92576 × 10^−5^
	q_e_	108.36312 ± 4.08751
	R^2^	0.97633
20 ppm	$k_{2}$	4.03958 × 10^−4^ ± 1.64286 × 10^−4^
	q_e_	103.71385 ± 8.88065
	R^2^	0.92994
**Elovich Kinetic model: ** $Y=\frac{1}{\beta }\ln(\alpha \beta X+1)$		
5 ppm	Adsorption rate at 0 min = α (mg/min)	11.93295 ± 5.97565
	The extent of surface coverage = β (g/mg)	0.03643 ± 0.00759
	Correlation Coefficient = R^2^	0.92883
10 ppm	α	10.91733 ± 3.61829
	β	0.04217 ± 0.00532
	R^2^	0.96147
20 ppm	α	6.42892 ± 3.18597
	β	0.03914 ± 0.00897
	R^2^	0.89915

## Data Availability

The data is available on reasonable request from the corresponding author.

## Supplementary Materials

The following supporting information can be downloaded at: https://www.mdpi.com/article/10.3390/nano12213778/s1, Figure S1: Reusability test of the of SERS sensor for ten cycles provided with a table for the descriptive statistics of the results.
