# Supplementary material for: In-Situ SERS Detection of Hg2+/Cd2+ and Congo Red Adsorption Using Spiral CNTs/Brass Nails
Source: Nanomaterials (Basel). 2022 Oct 26;12(21):3778. doi: 10.3390/nano12213778 (PMC9653861; doi:10.3390/nano12213778)
Supplement: Supplementary file 1 [file nanomaterials-12-03778-s001.zip › nanomaterials-1979805-supplementary.pdf]

In-Situ SERS Detection of Hg^{2+}/Cd^{2+} and Congo Red Adsorption Using Spiral CNTs/Brass Nails

Mohamed Shaban

Department of Physics, Faculty of Science, Islamic University of Madinah, Madinah 42351, Saudi Arabia; mssfadel@aucegypt.edu

N total	Mean	Standard Deviation	Sum	Minimum	Median	Maximum
10	994.42679	4.6917	9944.26791	986.31142	994.62234	1000

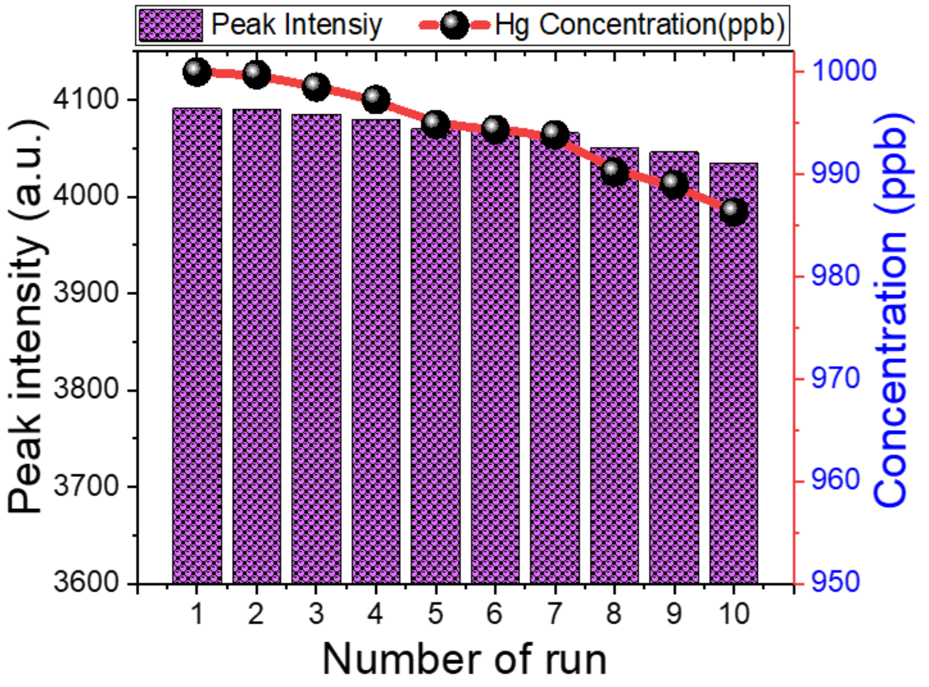

Figure S1. Reusability test of the of SERS sensor for ten cycles provided with a table for the descriptive statistics of the results. After each cycle, the SERS sensor is cleaned by rinsing with deionized (DI) water and drying with nitrogen.
